# Effects of Environmental Air Pollution on Pulmonary Function Level of Residents in Korean Industrial Complexes

**DOI:** 10.3390/ijerph15050834

**Published:** 2018-04-24

**Authors:** Eunju Hong, Seokwon Lee, Geun-Bae Kim, Tae-Jong Kim, Hyoung-Wook Kim, Kyoungho Lee, Bu-Soon Son

**Affiliations:** 1 Department of Environmental Health Science, College of Natural Science, Soonchunhyang University, 22 Soonchunhyang-ro, Asan City, Chungnam 31538, Korea; starsori98@nate.com (E.H.); hwpio88@naver.com (H.-W.K.); 2 Samsung Health Research Institute, Samsung Electronics Co. Ltd., 1 Samsungjeonja-ro, Hwaseong City, Gyeonggi-do 18448, Korea; sukwon23@gmail.com; 3 Environmental Health Research Division, National Institute of Environment Research, 42 Hwangyeong-ro, Incheon City 22689, Korea; nierkgb@naver.com; 4 World Vertex Corp., ACE bldg., 401m 179, Baumoe-ro, Seocho-gu, Seoul 06744, Korea; ceo@vtex.co.kr

**Keywords:** air pollution, health effect, industrial complex, ozone, pulmonary function test, sulfur dioxide

## Abstract

This study aims to identify environmental air pollution adversely affecting pulmonary function among a community-based general population living in Korean industrial complexes. A total of 1963 residents participated in a pulmonary function test (PFT). The sample population consisted of an exposed group (*n* = 1487) living within a radius of 5 km of industrial complexes and a control group (*n* = 476) living over a radius of 10 km from the industrial complexes in Gwangyang and Yeosu cities. PFT results were calculated for each resident of the study population. On-site questionnaire surveys with face-to-face interviews were also conducted to collect more detailed information on personal lifestyles, medical history, exposure to air pollution, and respiratory disease and related symptoms. A total of 486 measured samples were collected by eight automated air-monitoring stations installed in four counties of Gwangyang and four counties of Yeosu in South Korea from January 2006 to February 2007. Mean levels of SO_2_ (0.012 ppm), CO (0.648 ppm), NO_2_ (0.02 ppm), O_3_ (0.034 ppm), and PM_10_ (43.07 μg/m^3^), collected within a radius of 5 km, were significantly higher than those collected over a radius of 10 km from Gwangyang and Yeosu industrial complexes. Prevalence odds ratio (OR) of abnormal pulmonary function in the exposed group of residents (<5 km) was elevated at 1.24 (95% CI 0.71–1.96), but not statistically significant (*p* > 0.05). In multiple linear regression analysis, forced expiratory volume in one second (FEV_1_) and forced vital capacity (FVC) levels significantly declined as SO_2_, CO, and O_3_ levels increased when adjusting for age, sex, body mass index (BMI), alcohol, smoking, secondhand smoke, and respiratory disease and related symptoms (*n* = 1963) (*p* < 0.05). These results suggest that exposure to air pollution affects pulmonary function levels of residents living in Korean industrial complexes.

## 1. Introduction

Since the 1970s, a number of large-sized industrial complexes of petrochemical and steel industries were constructed and contributed to the fast economic growth in South Korea [1]. The industrial complexes were largely responsible for increasing serious problems, including various types of environmental pollution (such as stench, air pollution, soil contamination, and discharge of toxic metals into the surface water and groundwater). Huge concerns, including arguments from the government and local communities, have been raised from different public health perspectives [2,3]. Environmental pollution poses a huge threat to the well-being of human health in the general population of residents, and the standardized incidence or mortality ratios of environmental diseases emerged as one of the priorities of the national environmental health policy [4].

Since the early 2000s, the Ministry of Environment (MoE), one of the Korean government agencies, has rigorously reinforced environmental policy laws and regulations, which required installation of real-time air-monitoring stations fixed at each representative area of cities adjacent industrial complexes and monitoring the levels of exposure to environmental pollutants among general and susceptible populations [5]. As various environmental diseases with some related symptoms increased among polluted areas with residents living in several industrial complexes in Gwangyang, Sihwa, Ulsan, and Pohang in Korea, several environmental health studies were initiated with funds given by MoE. The research was conducted to investigate the association between exposure to air pollution and health effects among the local residents, including the general and susceptible populations in Korea [6,7,8].

Gwangyang (34°56′ N 127°41′′ E) Bay is one of the largest industrial complexes located in South Korea, with a population of approximately 150,000 and a size of 463.12 km^2^. The bay also includes both Yeosu petrochemical companies and Hadong thermal power plants. This industrial complex was rapidly developed during the past 35 years. Yeosu (34°44′ N 127°44′′ E) is another big industrial city with a population of about 300,000 and a size of 503.33 km^2^. It is located near the Gwangyang industrial complex, which is populated by large oil refineries and petrochemical manufacturing plants [9,10]. A number of studies have been conducted and reported that the levels of environmental air pollution—including nitrogen dioxide (NO_2_), carbon monoxide (CO), ozone (O_3_), dioxins, toxic metals, volatile organic compounds (VOCs), formaldehyde, polychlorinated biphenyls (PCBs), polycyclic aromatic hydrocarbons (PAHs), and particulate matter (PM)—were significantly increasing in the atmosphere, soil, and aquatic environments in these industrial complexes, which have been most affected [11,12,13]. Several studies concluded that chronic exposure to low levels of hazardous pollutants were significantly related to adverse effects on respiratory and cardiovascular systems among the residents living closer to Gwangyang and Yeosu industrial complexes in Korea [12,14].

There have been similar studies identifying an association between long-term exposure to air pollution and adverse effects on respiratory function, diseases, and allergy symptoms in the general population. The exposure to low levels of air pollution increases acute respiratory diseases, lung malfunction, blood pressure, and prevalence of respiratory diseases, with more frequent hospitalization. It exaggerates chronic respiratory diseases and increases the prevalence of asthma and asthma attacks [15,16,17]. Other studies have also shown that increased levels of exposure to air pollution (O_3_ and PM) were significantly associated with a high increase in risk in asthma among susceptible populations, including children, pregnant women, and elderly people [18,19,20,21]. In particularly, Bae et al. conducted a large-scale study about susceptible populations (i.e., school children) that would be the most vulnerable to the undesirable health effects due to long-term exposure to low levels of some pollutants (O_3_, NO_2_, PM, etc.) [22,23].

In many studies conducted on environmental epidemiology, quantitative and qualitative approaches such as questionnaire surveys, pulmonary function test (PFT), chest X-ray, and quantitative analysis of environmental-monitoring data were applied to evaluate adverse health effects of various environmental factors. The PFT has been widely used for large-scale environmental health research since the 1990s because of its lower cost, hence higher reliability of the test results [24]. Quite a few studies recommended that the results of PFT should not be considered as absolute, but predictive estimates among the target population of interest. This is because PFT results vary by age, sex, health condition, and ethnicity [25,26,27]. Despite the limitations of PFT results, the test is still useful for identifying associations between exposure to air pollution and adverse effects on pulmonary function of residents living in Korean industrial complexes.

Therefore, we conducted PFTs and on-site questionnaire surveys with face-to-face interviews and collected more detailed information on general characteristics of personal lifestyles, medical history, respiratory disease, allergic symptoms, and related environmental factors in a large community-based population consisting of individuals either (1) living within a radius of 5 km of Gwangyang and Yeosu industrial complexes or (2) living over 10 km from Gwangyang and Yeosu industrial complexes in Korea. The objective of this study was to compare pulmonary function levels (FEV_1_, FVC, and FEV_1_/FVC) between the exposed group (≤5 km) and control group (>10 km) of the study population, to observe which environmental factor or air pollutant could adversely affect the lung function, and to determine the relationship between exposure to environmental air pollution and adverse effects on lung function among residents living in Korean industrial complexes.

## 2. Methods and Materials

### 2.1. Study Subject

**Figure 1:**
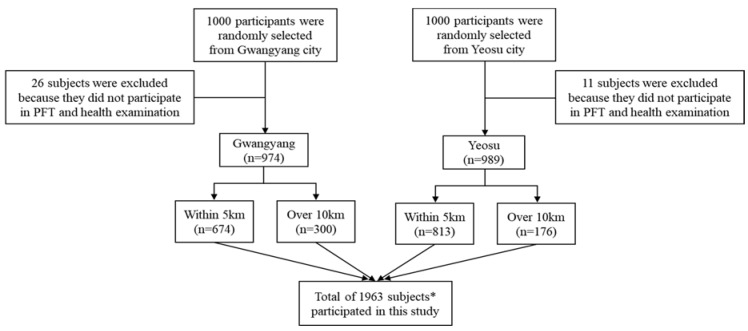
A diagram of selecting the study subjects (*n* = 1963) living in Gwangyang and Yeosu cities. * All study subjects were contacted by Ministry of Environment (MoE) via email or phone call, and MoE provided study information in advertisements on an MoE official website in 2007. PFT: pulmonary function test.

**Figure 2:**
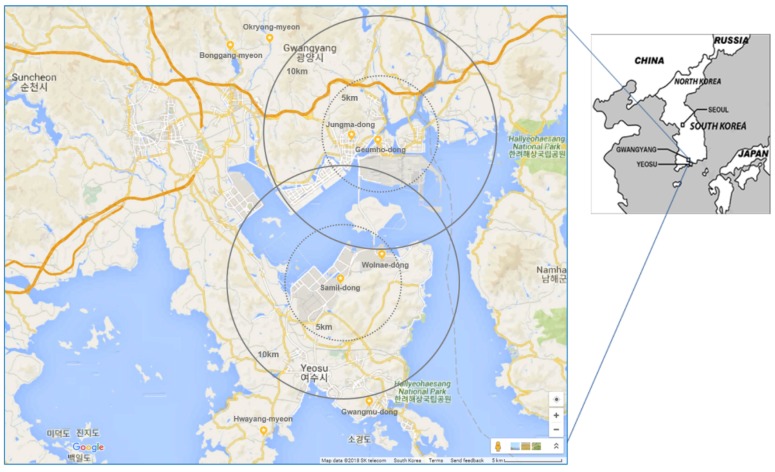
The map of study sites in Gwangyang and Yeosu industrial complexes in Korea.

This study was conducted by performing both PFTs and on-site questionnaire surveys for a total of 1963 residents from the general population who registered and lived in Gwangyang (*n* = 974) and Yeosu (*n* = 989) industrial complexes of Gwangyang Bay area in Korea, from May to December in 2007. We recruited a community-based population of people (ages from 5 to 70 years old) who had lived over 1 year in Gwangyang or Yeosu. All study subjects were contacted by MoE via email or phone call, and MoE provided information in advertisements on an MoE official website in 2007 (Figure 1). We enrolled up to 1000 persons who expressed interest in participating in the study. Consent was obtained from the study population by obtaining fully signed agreements to participate in PFTs, answer questionnaire surveys, and undergo medical examination, and for the study to make use of their personal information until the end of the study period. A total of 1963 participants were classified into exposed or control groups, depending on the distance of residence from the industrial complexes. Residents classified as exposed lived within a radius of 5 km from Gwangyang (*n* = 674) or Yeosu (*n* = 813) industrial complexes. The rest of residents were classified as a control group, who lived over a radius of 10 km from Gwangyang (*n* = 300) or Yeosu (*n* = 176) industrial complexes, respectively. Figure 2 shows the map of the study protocol. This study was approved by the Institutional Review Board (IRB) of Soonchunhyang University.

### 2.2. Questionnaire Survey

The study population (*n* = 1963) participated in the on-site questionnaire survey and provided their personal information (birth of data, sex, home address, etc.), socioeconomic status (education, occupation, income, etc.), personal lifestyle (smoking, alcohol consumption, exercise, etc.), residence (exposure to air pollution), nutrition (diet), time-activity pattern (daily, weekly, monthly), mental health (stress, attitude, personality, etc.), and medical history (respiratory disease, allergic symptoms, etc.). This survey was conducted during the summer season from July to August in the year 2007. The survey process was as follows. Several hard copies of questionnaires were provided to all participants at the meeting rooms at their local hospitals located in Gwangyang and Yeosu industrial complexes and several professional interviewers who completed preliminary training programs developed by Ministry of Environment (MoE) arrived at the meeting rooms. They asked some questions in response to answers the respondents gave regarding their personal opinions to gather additional information necessary to complete the on-site questionnaire surveys. The process took approximately 1 h to per each group of participants (about 7–10 subjects), including face-to-face interviews.

### 2.3. Pulmonary Function Test

Pulmonary function tests (PFTs) were performed on the entire study population using spirometers (Spirovit model SP1, Schiller, Ottobrunn, Germany) at national hospitals in both Gwangyang and Yeosu. Prior to PFTs, nurses who completed professional PFT programs offered by the Korean Occupational Health & Safety Agency (KOSHA) assisted in providing guidance for using the spirometers. The PFT results were calculated as forced expiratory volume in one second (FEV_1_), forced vital capacity (FVC), and FEV_1_/FVC after adjusting for age, sex, height, and weight from the Korean standard population [25]. The FVC—which determines the vital capacity of maximum respiratory function—was defined as an indicator of restrictive ventilatory impairment. FEV_1_ was defined as the volume which has been exhaled at the end of the first forced expiration and was used to evaluate obstructive ventilatory impairment. Finally, FEV_1_/FVC ratios were calculated to determine the category of pulmonary function. If the ratio was less than 0.70 (a golden standard [28]), then the predicted values of FEV_1_ and FVC were lower than 80% and obstructive lung disease (OLD) was diagnosed [29].

### 2.4. Collection of Air-Monitoring Data

A large number of air-monitoring datasets were collected by automated monitoring stations installed in four counties of Jungma-dong, Geumho-dong, Bonggang-myeon, and Okryong-myeon in Gwangyang city as well as four counties of Samil-dong, Wolnae-dong, Gwangmu-dong, and Hwayang-myeon in Yeosu city of Jeollanam-do in South Korea from January 2006 up to February 2007. A total of 486 measurement samples consisted of five pollutants: sulfur dioxide (SO_2_) (*n* = 97), carbon monoxide (CO) (*n* = 97), nitrogen dioxide (NO_2_) (*n* = 98), ozone (O_3_) (*n* = 97), and particulate matter (PM_10_) (*n* = 97). Legal environmental limits of all air pollutants are shown in Table 1. The values of arithmetic mean (AM) and standard deviation (SD) of each pollutant were statistically analyzed and compared for each area (Gwangyang and Yeosu). All collected monitoring data were used for statistical analysis in this study.

### 2.5. Statistical Analysis

All statistical analyses were performed using SAS 9.4 software package (SAS Inc., Cary, NC, USA) after completing data translation. Descriptive statistics were calculated for results of PFTs, exposure to ambient air pollutants, and on-site questionnaire surveys. Prevalence odds ratio (OR) with 95% confidence intervals (Cis) were also estimated to observe the effects of various environmental factors—including distance from industrial complexes, personal lifestyles (alcohol and smoking), respiratory disease and allergic symptoms—on pulmonary function among residents using a logistic regression model. Multiple regression analysis was also performed to identify how environmental factors (independent) significantly affected pulmonary function (dependent) among residents living near these industrial complexes after adjusting for age, sex, BMI, alcohol, smoking, and respiratory disease and related symptoms (e.g., asthma, wheezing, exercising wheezing, nasal obstruction, allergic rhinitis). As for multicollinearity, a collinearity analysis was performed to observe if there was any correlation between independent variables. Collinearity statistics (i.e., variance inflation factors, VIFs) were also calculated in multiple regression analysis. The null hypothesis was rejected in all statistical analyses if a *p*-value was less than the significance level (*p* < 0.05).

## 3. Results

Table 2 shows general characteristics of the individuals (from a community-based population) who participated in this study. Of 1963 subjects, female (*n* = 1164) residents accounted for about 60%, while school children (*n* = 639) aged from 10 to 19 years and the elderly (*n* = 599) aged over 60 years accounted for 32.6% and 30.5%, respectively. Non-response rate to the on-site questionnaire survey was 22.5% for secondhand smoke, 18% for smoking, and 10.3% for alcohol consumption.

Table 3 shows summary statistics of pulmonary function levels for all population groups in Gwangyang and Yeosu industrial complexes based on sex, age, distance from industrial complexes, body mass index (BMI), alcohol consumption, smoking, and secondhand smoke exposure. Overall, arithmetic means (Ams) and standard deviations (SDs) calculated for PFT results were 101.10 ± 23.30 for FEV_1_, 98.49 ± 19.42 for FVC, and 81.27 ± 9.27 for the ratio FEV_1_/FVC in Gwangyang, and 107.08 ± 23.68 for FEV_1_, 100.74 ± 19.27 for FVC, and 83.26 ± 9.62 for the ratio FEV_1_/FVC in Yeosu city. There was also a significant difference in risks to pulmonary function levels between exposed and control groups in both cities combined. All PFT values significantly decreased by age and personal lifestyles. Ratios of FEV_1_/FVC of nonsmokers were significantly higher than those of smokers in both cities (*p* < 0.05). Table 4 also shows summary statistics for levels of pulmonary functions (FEV_1_/FVC) in all groups of the study population (exposed, control, and total) classified by age. Pulmonary function levels in the exposed group were significantly lower than those in the control group in both areas (*p* < 0.05).

In Table 5, prevalence odds ratios (ORs) for abnormal pulmonary function in the exposed group were also elevated by some variables (including the distance from industrial complexes, distance from streets with automobiles, and exposure to secondhand smoke), but not statistically significant (*p* > 0.05). ORs were 1.24 (95% CI 0.71–1.96) for distance from industrial complexes (either ≤5 km or >10 km), 1.17 (95% CI 0.63–1.84) for distance from streets, 1.02 (95% CI 0.62–1.61) for alcohol, 0.92 (95% CI 0.49–1.72) for smoking, and 1.12 (95% CI 0.73–1.71) for secondhand smoke.

Levels of five air pollutants—SO_2_ (*n* = 97), CO (*n* = 97), NO_2_ (*n* = 98), O_3_ (*n* = 97), and PM_10_ (*n* = 97)—were automatically measured by eight fixed air-monitoring stations located in the counties of Gwangyang and Yeosu cities. The air monitoring data are summarized in Table 1. Overall, all levels of air pollutants (SO_2_, CO, NO_2_, O_3_, and PM_10_), which were regularly measured every month from January 2006 to February 2007, in the exposed area (≤5 km) were significantly higher than those in the control area (>10 km) (*p* < 0.05). All concentration levels of the five air pollutants were below the environmental limits established by MoE in 2007. Among these pollutants, CO showed the highest mean level. Mean levels of two pollutants (SO_2_ and O_3_) were significantly different between exposed and control areas in both cities (*p* < 0.05).

Table 6 shows results from multiple linear regression analysis, which was performed to observe if any pollutant was significantly associated with pulmonary function levels in each area. Pulmonary function of residents in both cities significantly declined as SO_2_, CO, and O_3_ levels increased (*p* < 0.05). Two pollutants (NO_2_ and PM_10_) were not related to pulmonary function levels in either area. No multicollinearity was observed between any variables in this regression model. All VIFs were less than 10 (data not shown, *p* < 0.05).

Table 7 shows that the increase of four pollutants (SO_2_, CO, O_3_, and PM_10_) was significantly associated with the decline in pulmonary function levels (FEV_1_, FVC, FEV_1_/FVC) in the exposed group (≤5 km) (adjusted r^2^ = 0.12) (*p* < 0.05). However, only two pollutants (SO_2_ and O_3_) were significantly related to the decline in lung function (FEV_1_ and FVC) in the control group, although the regression model for FEV_1_/FVC was not statistically significant. Most importantly, increased levels of O_3_ resulted in the highest decline in pulmonary function levels among the exposed group (population living within a radius of 5 km from Gwangyang and Yeosu industrial complexes in Korea).

## 4. Discussion

This study used pulmonary function tests with on-site questionnaire surveys to identify if environmental air pollution adversely affects pulmonary function levels in a community-based general population (*n* = 1963) living within 5 km (exposed) or over 10 km (control) from Gwangyang and Yeosu industrial complex areas located in South Korea. We observed that increased levels of SO_2_ and O_3_ were significantly associated with a decline in pulmonary function levels of residents living in the exposed area near industrial complexes. The Ams and SDs of PFTs in the exposed group were 99.17 ± 22.29 for FEV_1_ and 96.98 ± 18.86 for FVC in Gwangyang and 107.05 ± 23.52 for FEV_1_ and 100.23 ± 19.46 for FVC in Yeosu, respectively. Pulmonary function levels in the exposed group were significantly lower than those in the control group in all areas (*p* < 0.05).

In a multiple regression model, increased levels of SO_2_ and O_3_ were significantly associated with the decline in pulmonary function levels of residents in both Gwangyang and Yeosu cities when adjusting for age, sex, BMI, alcohol, smoking, secondhand smoke, and respiratory disease and related symptoms (adjusted r^2^ = 0.12). Our study results indicated that inhalation exposure to environmental air pollution, especially for SO_2_ and O_3_, was significantly associated with lung function problems in the general population living near Gwangyang and Yeosu industrial complexes. The increased level of ozone showed the biggest impact, causing a sharp decline in pulmonary function level in the susceptible population living within 5 km from Gwangyang industrial complex, indicating that environmental air pollution played a major role in abnormal pulmonary function.

Several similar studies have been previously reported over the years in Korea since the 1990s. Lee et al. conducted a study identifying that an environment exposed to SO_2_, O_3,_ and total suspended particulates (TSP) significantly increased mortality in the general population in Ulsan in Korea. The increased level of 100 μg/m^3^ of TSP was significantly related to elevated mortality ratios in the Seoul metropolitan city after adjusting for daily and seasonal variations in the exposure monitoring datasets [30]. In 2002, Kwon et al. reported that Asian dust events (CO, NO_2_, SO_2_, PM_10_, and O_3_) were significantly related to elevated risk of death from cardiovascular and respiratory diseases among the general population in Seoul during the period of 1995–1998 [31]. Son et al. conducted a study that showed an association between environmental air pollutants—which included CO, NO_2_, SO_2_, PM, and O_3_—with adverse effects on lungs using a large-scale population (*n* = 3827) recruited from Ulsan industrial complexes in Korea during the period of 2003–2007. The authors found that FVC levels were associated with all types of air pollutants. In particular, pulmonary functions declined by 6.1% for FVC and 0.5% for FEV_1_ as O_3_ level increased by 11 ppb [32].

Kim et al. conducted a cross-sectional study identifying an association between environmental air pollution and allergic diseases with a total of 4545 students living near industrial complexes in Korea. They also found that increased levels of black carbon, SO_2_, and O_3_ emitted from industrial complexes were significantly related to elevated ORs for allergic rhinitis and atopic dermatitis [33]. Smargiassi et al. investigated acute cardiorespiratory effects of air pollution (including SO_2_, NO_2_, PM_2.5_, benzene, and PAHs) for children aged 7–12 years (*n* = 72) with asthma living in proximity to an industrial complex area in Montreal, Canada. They found that a low level of personal exposure to benzene and PAHs was associated with decreased pulmonary function among children living in industrial complex areas [34]. In 2016, Wong et al. also conducted a cross-sectional study of long-term exposure to ambient air pollution, finding that it adversely affected children’s lung functioning in Canada from 2007 to 2011.

The authors found that industrial emission of PM_2.5_ was negatively associated with decreased lung functioning (FEV_1_ and FEV_1_/FVC) among young males, contrary to females [35]. An increase of 190 tons of industrial air emissions within 25 km of residence was associated with a 1% reduction in FEV_1_, and an increase of 370 tons was related to a 1% reduction in FEV_1_/FVC. Kurt et al. reviewed a total of 53 publications and observed how environmental air pollution is associated with health concerns related to pulmonary mobility and mortality worldwide. They suggested that air pollution can be an important factor when worsening lung diseases in susceptible populations, including children, pregnant women, and elderly people [36]. The environmental exposure to NO_X_, PM, and O_3_ was associated with elevated risk of asthma, chronic obstructive pulmonary disease (COPD), lung cancer, allergic symptoms, and respiratory infections at local, national, and global level [37]. PM and O_3_ were shown to be the most widespread health threats related to cardiopulmonary diseases. With regards to association between low-level exposure to ozone and pulmonary function, our study results showed great consistency with previous studies in Korea and other countries [38].

Our study has several strengths. First, we recruited a total of 1963 residents from a large-scale community-based population, aged between 5 and 70 years old, living in Gwangyang and Yeosu cities. All study participants, recruited from both Gwangyang and Yeosu cities, voluntarily participated in PFTs to measure their lung function levels and responded to questionnaire surveys regarding their personal lifestyles, distance from the industrial complexes, and respiratory disease and related symptoms. Secondly, the large sample population included susceptible individuals, including school children and elderly people. Approximately 70% of the study population consisted of school children and elderly people, while the rest of the population (30%) were young, aged from 20 to 59 years old. This is possibly due to a younger population working at both industrial complexes. Lastly, our study significantly identified an association between low-level exposure to air pollutants (SO_2_, CO, O_3_) and the decline in pulmonary function levels among the general and susceptible populations living in two Korean industrial complex areas.

Despite its strengths, this study had some limitations. The amount of air-monitoring data was relatively small without analyzing daily or seasonal variations, indicating that these collected datasets were not representative of true exposure to environmental air pollution in both industrial and residential areas. We only obtained a small number of air-monitoring data (*n* = 486) automatically collected by fixed monitoring stations located in eight counties in both Gwangyang and Yeosu cities. We did not collect personal monitoring data on daily, weekly, monthly, or seasonal bases. Furthermore, the average duration of each study participant’s (exposed or control group) outside daily activities was unknown because we did not collect detailed information on their daily activities or personal lifestyles (exposure misclassification). The exposed and control groups of the study population were only classified based on the distance of their registered home address from industrial complexes, as indicated on the self-administered questionnaire surveys and copies of written documents they submitted. Furthermore, we could not recruit enough numbers from the young population (aged from 20 to 39 years old). The young population might be working as industrial manufacturers or maintenance workers employed by either petrochemical or steel manufacturing plants located in both Gwangyang and Yeosu cities. Most importantly, we did not know where those five pollutants came from: we had no specific information as to whether these five pollutants were emitted from public or private transformation or industrial complex areas. Because we collected these five pollutants (SO_2_, CO, NO_2_, O_3_, and PM_10_) from MoE’s official website, we assumed that air pollution was mostly emitted from the target industrial complexes. Due to these limitations, further studies are needed to identify the relationship between environmental air pollution emitted from industrial complex areas in Gwangyang and Yeosu cites and adverse health effects on the most susceptible populations (school children and elderly) in the future.

Our study results might have shown no association between exposure to environmental air pollution and pulmonary function level in the community-based general population if more individuals from the young population group were included in this study. That is, the young population working in the industrial complexes must be much healthier than school children or elderly populations, particularly for lung function. They could show higher pulmonary function levels, although they were more likely to be exposed to higher levels of air pollutants or other hazardous chemicals emitted from their workplaces. Further studies will demonstrate if long-term exposure to environmental air pollutants significantly affects to pulmonary function levels in all age groups of the general and susceptible populations, including the young population, and these studies will also allow to identify which environmental factor adversely affects their respiratory and cardiovascular systems by investigating how these factors could result in the decline in lung function and other related symptoms among these residents. Finally, a new longitudinal study is also needed to determine how many cases would increase after exposure to low levels of environmental air pollution in the general and susceptible populations in Korea.

## 5. Conclusions

We found that there was an association between increased levels of exposure to ambient air pollutants (SO_2_, O_3_, and CO) and decline in pulmonary function levels (FEV_1_ and FVC) among residents living near Gwangyang and Yeosu industrial complexes in Korea. Risks for abnormal pulmonary function among residents were not statistically significant by independent variable. In this study, we found that increased levels of SO_2_ and O_3_ were significantly associated with the decline in pulmonary function levels of residents living in the exposed area near Korean industrial complexes.

## Figures and Tables

**Table 1:** The concentration levels of five air pollutants measured in both Gwangyang and Yeosu cities from January 2006 to February 2007.

Pollutant	Gwangyang (*n* = 277)							Yeosu (*n* = 209)							Total (*n* = 486)							Legal Limit ^∮^	Unit
	Exposed ^†^ (*n* = 138)			Control ^‡^ (*n* = 139)			*p-*Value	Exposed ^†^ (*n* = 140)			Control ^‡^ (*n* = 69)			*p-*Value	Exposed ^†^ (*n* = 278)			Control ^‡^ (*n* = 208)			*p-*Value		
	*n*	AM	SD	*n*	AM	SD		*n*	AM	SD	*n*	AM	SD		*n*	AM	SD	*n*	AM	SD			
SO_2_ (*n* = 97)	27	0.011	0.003	28	0.005	0.001	0.01 *	28	0.013	0.004	14	0.008	0.003	0.01 *	55	0.012	0.004	42	0.006	0.003	0.01 *	0.05	ppm
CO (*n* = 97)	28	0.739	0.249	27	0.443	0.112	0.00 *	28	0.557	0.233	14	0.535	0.198	0.77	56	0.648	0.256	41	0.478	0.151	0.00 *	9	ppm
NO_2_ (*n* = 98)	28	0.020	0.005	28	0.012	0.004	0.00 *	28	0.020	0.005	14	0.016	0.005	0.22	56	0.020	0.005	42	0.014	0.005	0.03 *	0.06	ppm
O_3_ (*n* = 97)	27	0.034	0.009	28	0.009	0.008	0.03 *	28	0.033	0.007	14	0.007	0.008	0.02 *	55	0.034	0.008	42	0.008	0.008	0.02 *	0.06	ppm
PM_10_ (*n* = 97)	28	39.39	7.20	28	36.50	11.74	0.27	28	46.75	13.00	13	35.31	12.05	0.00 *	56	43.07	11.06	41	36.12	11.70	0.00 *	100	μg/m^3^

* Statistically significant (*p*<0.05); ^†^ collected the data from air monitoring stations within a radius of 5 km; ^‡^ collected the data over a radius of 10 km; ^∮^ environmental limit values established by MoE in 2007.

**Table 2:** Characteristics of the community-based population in Gwangyang and Yeosu cities in Korea.

Variable	Gwangyang (*n* = 974) (%)	Yeosu (*n* = 989) (%)	Total (*n* = 1963) (%)
Sex			
Male	438 (45.0)	361 (36.5)	799 (40.7)
Female	536 (55.0)	628 (63.5)	1164 (59.3)
Age			
<9	18 (1.8)	49 (5.0)	67 (3.4)
10–19	325 (33.4)	314 (31.7)	639 (32.6)
20–39	70 (7.2)	87 (8.8)	157 (8.0)
40–59	308 (31.6)	193 (19.5)	501 (25.5)
>60	253 (26.0)	346 (35.0)	599 (30.5)
Distance from industrial complexes			
≤5 km ^†^	674 (69.2)	813 (82.2)	1487 (75.8)
>10 km ^‡^	300 (30.8)	176 (17.8)	476 (24.2)
BMI			
<18.5	169 (17.4)	224 (22.6)	393 (20.0)
18.5–22.9	593 (60.9)	555 (56.1)	1148 (58.5)
23–24.9	190 (19.5)	183 (18.5)	373 (19.0)
>25	22 (2.3)	27 (2.7)	49 (2.5)
Alcohol *			
No	607 (62.3)	618 (62.5)	1225 (62.4)
Yes	303 (31.1)	233 (23.6)	536 (27.3)
Smoking *			
No	684 (70.2)	696 (70.4)	1380 (70.3)
Yes	150 (15.4)	79 (8.0)	229 (11.7)
Secondhand smoke *			
No	426 (43.7)	372 (37.6)	798 (40.7)
Yes	356 (36.6)	366 (37.0)	722 (36.8)

^†^ Exposed group living within a radius of 5 km from industrial complexes; ^‡^ control living over a radius of 10 km; * some subjects did not respond to the survey question.

**Table 3:** Summary statistics of pulmonary function levels of all residents with general characteristics, personal lifestyle, and air pollution.

Variable	Gwangyang (*n* = 974)							Yeosu (*n* = 989)							Total (*n* = 1963)						
	*n*	FEV_1_		FVC		FEV_1_/FVC		*n*	FEV_1_		FVC		FEV_1_/FVC		*n*	FEV_1_		FVC		FEV_1_/FVC	
		AM	SD	AM	SD	AM	SD		AM	SD	AM	SD	AM	SD		AM	SD	AM	SD	AM	SD
Total	974	101.10	23.30	98.49	19.42	81.27	9.27	989	107.08	23.68	100.74	19.27	83.26	9.62	1963	104.91	24.83	101.60	20.82	81.80	9.84
Sex																					
Male	438	99.35	21.15	99.64	19.29	80.37	9.10	361	103.38	20.78	101.64	17.30	81.56	10.55	799	101.26	21.26	100.58	18.36	80.98	9.79
Female	536	102.55	24.91	97.56	19.52	81.59	9.35	628	109.21	24.96	100.22	20.30	84.24	8.91	1164	105.67	24.84	99.76	20.18	82.79	9.16
*p*-value		0.03 *		0.10		0.23			0.01 *		0.26		0.01 *			0.01 *		0.35		0.14	
Distance from industrial complexes																					
>10 km ^†^	300	105.47	25.05	101.91	20.26	81.41	8.48	176	107.26	24.47	102.85	18.24	83.67	9.65	476	107.25	28.33	104.89	22.90	81.10	10.14
≤5 km ^‡^	674	99.17	22.29	96.98	18.86	80.97	9.59	813	107.05	23.52	100.28	19.46	83.17	9.62	1487	103.95	23.17	100.23	19.74	82.09	9.70
*p*-value		0.01 *		0.01 *		0.11			0.92		0.10		0.54			0.03 *		0.01 *		0.40	
BMI (kg/m^2^)																					
<18.5	169	89.77	17.62	94.06	16.57	84.37	8.87	224	95.40	17.68	96.79	17.16	86.23	9.42	393	92.34	17.64	95.26	16.97	85.46	9.12
18.5–22.9	593	102.88	23.78	99.36	19.60	80.59	9.76	555	109.71	24.76	101.49	18.97	82.52	9.83	1148	105.69	24.19	100.69	19.10	81.34	9.79
23–24.9	190	105.66	23.75	99.65	20.09	80.67	7.46	183	112.41	22.30	102.24	19.49	82.33	8.54	373	107.97	23.06	101.26	19.74	81.46	8.04
>25	22	101.38	20.78	99.29	25.05	81.14	8.61	27	113.87	23.09	107.69	32.18	80.24	9.21	49	108.46	21.97	103.45	28.95	80.68	8.89
*p*-value		0.01 *		0.01 *		0.01 *			0.01 *		0.01 *		0.01 *			0.01 *		0.01 *		0.01 *	
Alcohol																					
No	607	102.34	22.15	98.99	20.87	81.82	9.50	618	109.79	23.66	100.67	20.05	83.78	9.28	1225	105.54	21.24	99.46	20.43	81.41	9.42
Yes	303	100.47	24.22	98.06	18.91	79.95	8.63	233	107.52	23.74	100.99	19.34	81.37	10.69	536	103.76	22.63	99.68	19.04	80.57	9.56
*p*-value		0.24		0.50		0.01 *			0.22		0.83		0.01 *			0.01 *		0.78		0.55	
Smoking																					
No	684	101.16	22.01	99.18	20.04	79.17	9.19	696	109.09	24.45	101.56	16.41	78.69	11.39	1380	105.36	23.16	100.23	18.64	78.98	10.46
Yes	150	100.98	24.21	98.30	19.91	81.41	9.35	79	107.28	21.80	100.86	19.81	83.77	9.47	229	104.14	22.33	99.84	21.42	82.59	9.41
*p*-value		0.93		0.63		0.01 *			0.49		0.73		0.01 *			0.36		0.43		0.01 *	
Secondhand Smoke																					
No	356	105.48	25.26	99.42	20.25	80.39	9.97	372	112.09	26.52	98.93	20.39	81.15	9.42	728	108.59	25.10	99.12	20.31	80.98	9.79
Yes	426	97.25	22.44	98.53	19.88	81.77	8.86	366	105.29	21.08	102.43	18.64	83.16	10.05	792	103.46	21.89	100.21	19.26	82.36	9.58
*p*-value		0.01 *		0.54		0.04 *			0.01 *		0.16		0.45			0.01 *		0.39		0.17	

* Statistically significant (*p* < 0.05); ^†^ control group living over a radius of 10 km from industrial complexes; ^‡^ exposed group living within a radius of 5 km; FEV_1_: forced expiratory volume in one second; FVC: forced vital capacity; AM: arithmetic mean; SD: standard deviation; BMI: body mass index.

**Table 4:** Summary statistics of pulmonary function levels of distance from industrial complexes stratified by age.

Variable	Gwangyang (*n* = 974)							Yeosu (*n* = 989)							Total (*n* = 1963)						
	*n*	FEV_1_		FVC		FEV_1_/FVC		*n*	FEV_1_		FVC		FEV_1_/FVC		*n*	FEV_1_		FVC		FEV_1_/FVC	
		AM	SD	AM	SD	AM	SD		AM	SD	AM	SD	AM	SD		AM	SD	AM	SD	AM	SD
Within a radius of 5 km from industrial complexes																					
<9	18	98.50	12.01	102.72	12.76	86.59	7.97	38	96.84	12.48	99.32	13.16	87.80	7.88	56	97.38	12.24	100.41	13.02	87.41	7.64
10–19	218	89.54	15.23	97.14	16.05	84.56	7.77	238	96.49	15.23	100.70	17.16	85.59	8.87	456	93.17	15.61	99.00	16.71	85.10	8.37
20–39	41	100.87	20.19	97.85	21.33	82.47	8.40	84	100.54	15.54	95.26	13.84	83.94	6.69	125	100.65	17.18	96.11	16.63	83.46	7.30
40–59	224	105.85	19.32	98.63	16.16	80.69	7.71	161	111.99	20.40	102.96	22.02	82.35	9.08	385	108.42	19.98	100.44	18.93	81.49	8.33
>60	173	102.33	29.52	93.82	24.28	75.63	11.63	292	116.13	28.69	100.02	21.50	80.84	10.74	465	110.99	29.73	97.71	22.74	78.90	11.34
Over a radius of 10 km from industrial complexes																					
<9	-	-	-	-	-	-	-	11	90.82	8.82	93.91	10.01	86.65	7.62	11	90.82	8.83	93.91	10.01	86.65	7.62
10–19	107	92.89	17.37	100.03	18.86	83.58	8.54	79	97.26	15.44	101.41	13.85	87.45	7.56	183	94.71	16.69	100.60	16.94	85.18	8.35
20–39	29	100.40	18.89	94.54	15.31	84.63	8.36	3	118.67	17.93	111.67	9.50	82.87	7.18	32	102.07	19.00	97.53	17.98	83.29	7.41
40–59	84	112.11	23.55	104.55	18.58	81.52	6.71	32	119.13	18.27	107.81	15.26	82.24	5.28	116	114.05	22.37	105.45	17.72	81.72	6.34
>60	80	117.17	29.19	104.33	24.32	79.24	9.40	54	117.00	32.07	103.26	25.08	78.63	12.16	134	117.11	30.27	103.89	24.54	78.99	10.56
Total																					
<9	18	98.50	12.01	102.72	12.76	86.59	7.97	49	95.49	11.94	98.10	12.64	87.54	7.51	67	96.30	11.95	99.34	12.74	87.29	7.58
10–19	325	90.65	16.02	98.09	17.05	84.24	8.03	314	96.68	15.27	100.87	16.40	86.04	8.59	639	93.60	15.93	99.46	16.78	85.12	8.35
20–39	70	100.68	19.52	96.48	19.01	83.37	8.40	87	101.17	15.95	95.83	14.01	83.90	6.67	157	100.95	17.58	96.12	16.37	83.66	7.47
40–59	308	107.56	20.71	100.25	17.03	81.06	7.45	193	113.18	20.19	103.77	21.10	82.33	8.56	501	109.72	20.68	101.60	18.76	81.55	7.91
>60	253	107.02	30.16	97.14	24.74	76.77	10.11	346	116.26	29.19	100.53	22.09	80.50	10.98	599	112.36	29.93	99.09	23.28	78.92	11.17
*p*-value		0.00 *		0.24		0.00 *			0.00 *		0.02 *		0.00 *			0.00 *		0.03 *		0.00 *	

* Statistically significant (*p* < 0.05).

**Table 5:** Prevalence odds ratios (OR) of abnormal pulmonary functions of residents in both cities calculated by logistic regression analysis.

Variable	Total (*n* = 1963)				
	*n*	Abnormal Pulmonary Functions		Adjusted OR ^†^	95% CI
		Yes	No		
Sex					
Female	799	101	698	Reference	-
Male	1164	145	1019	0.79	0.54–1.17
Distance from industrial complexes					
>10 km	476	59	417	Reference	-
≤5 km	1487	187	1300	1.24	0.71–1.96
Distance from the streets					
>100 m	1222	128	1094	Reference	-
≤100 m	332	42	290	1.15	0.63–1.84
Alcohol					
No	1225	167	1058	Reference	-
Yes	536	54	482	1.02	0.62–1.61
Smoking					
No	1380	175	1205	Reference	-
Yes	229	29	200	0.92	0.49–1.72
Secondhand smoke					
No	728	84	644	Reference	-
Yes	792	110	682	1.12	0.73–1.71

^†^ The model was adjusted for age, education, BMI, and respiratory disease and related symptoms (e.g., asthma, wheezing, exercising wheezing, nasal obstruction, allergic rhinitis).

**Table 6:** Multiple linear regression analysis for pulmonary function levels by air pollutant in each area.

Pollutant	Gwangyang (*n* = 974)									Yeosu (*n* = 989)									Total (*n* = 1963)								
	FEV_1_ (r^2^ = 0.65)			FVC (r^2^ = 0.61)			FEV_1_/FVC (r^2^ = 0.15)			FEV_1_ (r^2^ = 0.33)			FVC (r^2^ = 0.34)			FEV_1_/FVC (r^2^ = 0.13)			FEV_1_ (r^2^ = 0.48)			FVC (r^2^ = 0.48)			FEV_1_/FVC (r^2^ = 0.12)		
	β	SE	*p-*Value	β	SE	*p-*Value	β	SE	*p-*Value	β	SE	*p-*Value	β	SE	*p-*Value	β	SE	*p-*Value	β	SE	*p*-Value	β	SE	*p-*Value	β	SE	*p-*Value
SO_2_	−22.63	5.17	0.00 *	−28.54	6.25	0.00 *	−18.27	7.07	0.03 *	−18.85	4.94	0.00 *	−20.12	5.84	0.00 *	−10.82	8.32	0.47	−17.43	3.44	0.00 *	−20.80	4.14	0.00 *	−12.73	6.85	0.04 *
CO	−0.27	0.10	0.01 *	−0.35	0.12	0.02 *	−0.17	1.46	0.91	−0.12	0.09	0.20	−0.18	0.11	0.10	−0.04	1.26	0.50	−0.15	0.07	0.02 *	−0.23	0.08	0.00 *	−0.08	1.07	0.48
NO_2_	5.49	4.77	0.25	7.68	5.76	0.18	8.23	9.64	0.92	2.78	4.47	0.54	2.54	5.28	0.63	5.69	7.44	0.48	5.27	3.08	0.09	6.06	3.72	0.08	7.82	5.10	0.73
O_3_	−43.00	2.21	0.00 *	−46.89	2.81	0.00 *	−21.19	4.04	0.00 *	−16.42	2.17	0.01 *	−19.73	2.62	0.01 *	−13.49	3.68	0.02 *	−24.91	1.53	0.00 *	−24.85	2.75	0.00 *	−17.87	2.54	0.04 *
PM_10_	−0.04	0.03	0.35	−0.01	0.02	0.03 *	−0.02	0.03	0.48	0.01	0.02	0.61	0.01	0.02	0.62	0.01	0.03	0.70	0.02	0.01	0.17	0.03	0.01	0.16	−0.01	0.02	0.96

* Statistically significant (*p* < 0.05); the regression model was adjusted for age, sex, BMI, smoking, secondhand smoke, alcohol, and respiratory disease and related symptoms (e.g., asthma, wheezing, exercising wheezing, nasal obstruction, allergic rhinitis); no multicollinearity for independent variables was observed (all variance inflation factors (VIFs) < 10).

**Table 7:** Multiple linear regression analysis for pulmonary function levels by air pollutant in each group classified by distance from industrial complexes.

Pollutant	Within a Radius of 5 km (Exposed) (*n* = 1487)									Over a Radius of 10 km (Control) (*n* = 476)								
	FEV_1_ (r^2^ = 0.50)			FVC (r^2^ = 0.48)			FEV_1_/FVC (r^2^ = 0.12)			FEV_1_ (r^2^ = 0.51)			FVC (r^2^ = 0.51)			FEV_1_/FVC (r^2^ = 0.11)		
	β	SE	*p-*Value	β	SE	*p-*Value	β	SE	*p-*Value	β	SE	*p-*Value	β	SE	*p-*Value	β	SE	*p-*Value
SO_2_	−18.17	4.07	0.00 *	−22.53	4.93	0.00 *	−19.33	7.02	0.04 *	−16.45	6.29	0.01 *	−17.48	7.47	0.02 *	−11.11	9.55	0.59
CO	−0.19	0.08	0.01 *	−0.30	0.09	0.00 *	−0.15	1.30	0.26	−0.06	0.12	0.61	−0.06	0.15	0.71	−0.14	0.19	0.55
NO_2_	7.25	3.55	0.04 *	7.64	4.30	0.05 *	7.07	6.13	0.27	2.37	6.14	0.70	0.61	7.29	0.93	−1.29	9.32	0.67
O_3_	−17.43	1.78	0.00 *	−20.57	2.16	0.00 *	−45.95	3.07	0.00 *	−20.62	2.99	0.00 *	−22.56	3.55	0.00 *	−19.42	4.54	0.17
PM_10_	−0.02	0.01	0.02 *	−0.01	0.01	0.02 *	−0.02	0.02	0.24	0.01	0.01	0.34	0.01	0.02	0.34	−0.01	0.04	0.85

* Statistically significant (*p* < 0.05), ^†^ the regression model was adjusted for age, sex, BMI, alcohol, smoking, secondhand smoke, and respiratory disease and related symptoms (e.g., asthma, wheezing, exercising wheezing, nasal obstruction, allergic rhinitis); no multicollinearity for independent variables was observed (all VIFs < 10).
